# Infectious disease forecasting to support public health: use of readily available methods to predict malaria and diarrhoeal diseases in Mozambique

**DOI:** 10.7189/jogh.15.04114

**Published:** 2025-04-11

**Authors:** Rami Yaari, Marta Galanti, Rodrigo Zepeda-Tello, Sergio Chicumbe, Ilesh Jani, Annette Cassy, Ivalda Macicame, Naisa Manafe, Shannon M Farley, Wafaa M El-Sadr, Jeffrey Shaman

**Affiliations:** 1 Mailman School of Public Health, Columbia University, New York, New York, USA; 2 National Health Institute of Mozambique (Instituto Nacional de Saúde, INS), Maputo, Mozambique; 3 Ministry of Health, Maputo, Mozambique; 4 ICAP, Columbia University, New York, New York, USA; 5 Climate School, Columbia University, New York, New York, USA

## Abstract

**Background:**

Mozambique faces a high burden of infectious diseases but currently has limited capacity for forecasting disease incidence. Recent improvements in disease surveillance through the National Monitoring and Evaluation System now provide weekly reports of disease incidence across the country’s districts. This study focuses on using these records, specifically for malaria and diarrhoeal diseases, which together account for approximately 40% of deaths among children under five, to develop statistical forecasts and evaluate their accuracy.

**Methods:**

We utilised a Python library for time series forecasting called Darts, which includes a variety of statistical forecasting models. Three models were selected for this analysis: Exponential Smoothing (a classical statistical model), Light Gradient Boosting Machine (a machine-learning model), and Neural Hierarchical Interpolation for Time Series (a neural network-based model). Retrospective forecasts were generated and compared across multiple forecast horizons. We evaluated both point and probabilistic forecast accuracy for individual models and two types of model ensembles, comparing the results to forecasts based on historical expectance.

**Results:**

All models consistently outperformed forecasts based on historical expectance for both malaria and diarrhoeal disease across forecast horizons of up to eight weeks, with comparable or better performance at 16 weeks. The most accurate forecasts were achieved using a weighted ensemble of the models.

**Conclusions:**

This study highlights the potential of using a readily available tool for generating accurate disease forecasts. It represents a step toward scalable and accessible forecasting solutions that can enhance disease surveillance and public health responses, not only in Mozambique but also in other low- and middle-income countries with similar challenges.

Mozambique is one of the world's least resourced nations, positioned at 185 out of 191 countries on the Human Development Index in 2021 [1]. The country grapples with numerous challenges, including weak infrastructure and limited access to clean water, sanitation, roads, and health facilities [2]. In addition, the country is persistently confronted with natural disasters, including cyclones, droughts, and flooding, as well as ongoing conflict, particularly in its northern provinces [3]. Consequently, Mozambique faces a multitude of health issues, ranging from chronic non-communicable diseases like heart disease, malnutrition, and birth defects, to infectious diseases such as human immunodeficiency virus, tuberculosis, malaria, and diarrhoeal diseases. The latter two, malaria and diarrhoeal diseases, are prevalent nationwide and jointly contribute to a significant portion, approximately 40%, of deaths among children under five years of age [4].

The entire country of Mozambique is endemic for malaria. It is among the four nations, along with Nigeria, Democratic Republic of Congo, and Uganda, responsible for over half of global malaria deaths [5]. Despite concerted efforts by the National Malaria Control Programme and international consortia including the United States Agency for International Development and the United Nations Children's Fund, progress in reducing the malaria disease burden, notable in the early 2000s, has plateaued in the last decade, coincident with an increase in malaria cases noted across Sub-Saharan Africa [6]. The complex interplay of factors contributing to this trend include changes in climate, stagnation in infrastructure and interventions implementation, and the adaptive resistance of vectors to insecticides [7].

Diarrhoeal diseases arise from various enteric pathogens, including bacteria such as *E coli*, *Salmonella, Shigella, Vibrio cholerae,* and *Campylobacter*; viruses like rotavirus, norovirus, adenovirus, and astrovirus; and parasites including *Cryptosporidium*, *Giardia lamblia*, and *Entamoeba histolytica* [8]. Despite a declining trend over the past decades [9], diarrhoeal diseases persist as a significant cause of morbidity and mortality among children under five in sub–Saharan Africa and South Asia. Factors contributing to the global burden of diarrhoeal diseases include limited access to safe drinking water, inadequate sanitation, and poor hygiene practices [10]. Malnutrition, restricted health care access, especially limited availability of oral rehydration solutions, and co-occurrence with immune-compromising diseases like human immunodeficiency virus are all key factors influencing mortality associated with diarrhoeal diseases [11].

Statistical and mathematical models have gained widespread use globally, successfully replicating patterns across various disease systems, such as influenza [12,13], malaria [14,15], and SARS-CoV-2 [16,17]. The recent COVID-19 pandemic has demonstrated that such methods can be incorporated into the public health system to guide the response to emerging and endemic diseases. When integrated with robust disease surveillance, these forecasting tools are invaluable assets for decision-making and resource allocation to ensure that effective preventive and treatment measures are deployed promptly where needed. This is particularly valid in low-income countries, where resources are typically limited and need to be allocated efficiently [18].

Despite significant progress in disease surveillance, Mozambique currently lacks accessible and scalable forecasting tools that can be seamlessly integrated into routine public health operations. While some predictive models for malaria have been previously published [19,20], these are typically limited to individual districts and focus on specific processes, such as links to climatic factors, rather than providing generalised, operational tools. This study aims to bridge this gap by adapting and testing a suite of statistical forecasting methods, packaged into a general, practical and user-friendly tool designed to enable real-time forecasting of both malaria and diarrhoeal diseases with minimal computational requirements. To achieve this, we leveraged approaches implemented within a specialised Python library for time series forecasting, Darts [21]. Several types of statistical models were evaluated, including a traditional statistical model, a machine-learning regression model, and a state-of-the-art neural network model. These models were applied to generate forecasts across varying horizons (two, four, eight, and 16 weeks) and geographical regions. The forecasts from individual models were then combined into ensemble forecasts using two distinct approaches. Finally, we conducted a comparative analysis of the accuracy of point and probabilistic forecasts among these models and against a baseline of historical expectance forecasts.

## METHODS

### Incidence data

Incidence data were extracted by the National Health Institute of Mozambique (Instituto Nacional de Saúde, INS) through the National Monitoring and Evaluation System (SIS-MA). The data set is comprised of weekly reports of confirmed malaria cases and diarrhoeal disease cases at the district level from January 2016 to December 2022 (365 weeks). Malaria cases were confirmed using either a positive rapid antigen test for *Plasmodium* species or by visual confirmation of the *Plasmodium* parasite via optic microscopy of a blood smear stained with Giemsa. Diarrhoeal diseases cases were defined based on symptoms and stool frequency, and characteristics. Due to poor sanitation, water security, and food security, diarrhoeal diseases cases are all syndromically classified as infectious diarrhoea.

The district level data were aggregated to the province level for the 11 administrative provinces of Mozambique: Niassa, Cabo Delgado, Tete, Nampula, Sofala, Zambezia, Manica, Inhambane, Tete, Maputo and Maputo City. Weekly incidence rates for each province were calculated, accounting for the population coverage of reports each week. The population coverage was determined as the percentage of the population in reporting districts within each province for the respective week (Figure S1 in the **Online Supplementary Document**). We employed seasonal and trend decomposition using moving averages to extract the trend and seasonality patterns of each of the weekly incidence time series. Age-group incidence data were available for part of the study period at a monthly rate. It was used to calculate the proportion of children under five among total cases in the different provinces.

### Demographics data

Population sizes at the provincial level for the modelled period were extrapolated from projections derived from 2017 census data. To facilitate the calculation of weekly malaria and diarrhoeal diseases incidence rates for each province, we performed linear interpolation to convert the yearly data into a weekly format.

### The forecasting models

The Darts Python library was specifically developed for the purpose of forecasting and anomaly detection in time series data. Its suite of forecasting models includes a range of traditional statistical models as well as more advanced machine-learning regression models and PyTorch-based neural-network models. For this study, we employed three models that represent these three model types: Exponential Smoothing [22], a classical statistical model that decomposes a time series to a baseline, trend and seasonal components; Light Gradient Boosting Machine (LightGBM) [23], a machine-learning ensemble decision tree method designed for classification and regression tasks has been effectively adapted for time series forecasting; and Neural Hierarchical Interpolation for Time Series (N-HiTS) [24], a state-of-the-art neural-network architecture tailored for time series forecasting. These models were selected based on the following criteria:

1. reputation as robust and widely regarded methods in their respective categories

2. efficient runtime for the training procedure, ensuring applicability in resource-limited settings

3. support for probabilistic forecasting, which is important for evaluating uncertainty in predictions.

With our data sets, these three selected models displayed the fastest training runtime out of the models in their category, making them highly suitable for practical application. Sections A–B in the **Online Supplementary Document** provide more details on each model and its configuration.

### Forecast generation and validation

We employed a rolling-origin evaluation procedure [25] for evaluating the predictive capabilities of each of the selected models at forecasting malaria and diarrhoeal diseases incidence in Mozambique. In this approach, starting from the beginning of the time series and with an initial training set of a minimum required size, each model is used to generate predictions for the subsequent time steps up to the maximal required forecast horizon. The training set is then progressively expanded to incorporate the next data point, and the models are retrained using the extended training set to generate predictions for the subsequent time steps. The process concludes when the end of the time series is reached. For each tested forecast horizon, h, the forecasted time series, f_h_, is extracted from the generated predictions, such that f_h_(t) contains the forecast for time t generated at time t–h. The forecasted time series is then assessed against observed time series values. For our study, the minimum required training set size was set to two and a half years, initiating the first forecasts at the beginning of July 2018. We stress that in this approach, each forecast is generated using only the data available up to that point in time, with no access to future observations. This ensures that the evaluation reflects the models' actual forecasting capabilities in a realistic, real-time forecasting scenario, avoiding any lookahead bias and providing a rigorous assessment of their performance.

We examined the forecasting capabilities for four forecasting horizons of two, four, eight and 16 weeks. These horizons were chosen to evaluate the models’ performance over varying lead times, which is critical for supporting different types of public health decision-making. Short-term forecasts (*e.g*. two and four weeks) are valuable for immediate response planning, while longer-term forecasts (*e.g*. eight and 16 weeks) can inform strategic preparations and resource allocation for seasonal disease trends. All generated forecasts were probabilistic, yielding forecasts for 23 selected quantiles (Section C in the **Online Supplementary Document**). The models did not incorporate any external predictors (Section D in the **Online Supplementary Document**). This decision was made to simplify the forecasting process for end-users and ensure that the tool relies only on data consistently available through existing surveillance systems, avoiding potential complications from missing or delayed external data. The forecasts were evaluated using four different metrics: root mean squared error (RMSE), mean absolute percentage error (MAPE), symmetric mean absolute percentage error (SMAPE) and weighted interval score (WIS) – a metric for evaluating probabilistic forecasts [26] (Section E in the **Online Supplementary Document**). The results of the forecasting models were ensembled in two ways – a mean ensemble that averages the forecasts of the models, and a WIS-weighted ensemble that computes a weighted average using the WIS score of the models in the preceding weeks (Section F in the **Online Supplementary Document**). Ensembles are commonly used in forecasting because they often outperform individual models by leveraging the strengths of each model while mitigating their individual weaknesses, improving robustness and accuracy [12,17].

### Historical expectance forecasts

The forecasts of the tested models and their ensembles were compared to a baseline model of historical expectance in which the number of cases for a specific year and week is forecast based on the mean (x̄) number of cases during the same week in the previous years within a specified window. Employing a window size of one year corresponds to a naive seasonal model, which repeats the seasonal pattern of the previous year. To refine this baseline, we searched for the window size that produced the most accurate historical expectance forecasts and selected a window size of two years for this study. This baseline model was chosen not only for its simplicity but also because it reflects the forecasting approach currently accessible to public health officials in Mozambique, making it a practical and meaningful comparator for evaluating the tested models.

### Visualisation dashboard

To support the use of the forecasting tool by INS personnel, we developed a visualisation dashboard that automatically produces plots and summary statistics from the forecasted time series.

## RESULTS

### Incidence data analysis

Between 2016–2022, there were on average 600 000 cases of diarrhoeal diseases and 7.6 million cases of malaria per year in Mozambique. Figure S2 in the **Online Supplementary Document** shows the average incidence rates per 100 000 population for both conditions. Figure S3 in the **Online Supplementary Document** presents the weekly incidence rates alongside the historical expected projections, derived using a two-year window that provided the best fit for this model. Throughout the study period, malaria incidence was heterogeneous across the country, with average weekly cases ranging from 39 cases (Maputo City) to 820 cases (Inhambane) per 100 000 population. The heterogeneity of malaria incidence seems to have some geographical pattern, with higher rates in the northern and coastal provinces (Cabo Delgado, Niassa, Nampula, Zambezia, Sofala, Inhambane), and much lower rates in the southern provinces (Maputo, Maputo City). The trend of malaria incidence during the study period was mostly stable or increasing, except for the two southern provinces (Maputo, Maputo City) that showed a declining trend (Figure S4 in the **Online Supplementary Document**). Diarrhoeal diseases incidence was also heterogeneous, however without any clear geographical pattern, with average weekly cases ranging from 19 cases (Manica) to 73 cases (Niassa) per 100 000 population. Diarrhoeal diseases incidence demonstrated a declining trend in all provinces (Figure S5 in the **Online Supplementary Document**).

Both malaria and diarrhoeal diseases exhibit a seasonal pattern, with increased incidence associated with the rainy season, which spans from November through April. However, malaria seasonality seems more pronounced with a more heterogeneous peak timing compared to the seasonality of diarrhoeal diseases (Figure S6 in the **Online Supplementary Document**). The seasonal peak of malaria incidence ranges from the beginning of February (Maputo, Maputo City, Niassa) to the middle of May (Sofala, Gaza). The peak seasonality of diarrhoeal diseases ranges from the end of January (Tete, Manica, Niassa) to the middle of March (Maputo, Maputo City, Zambezia), often preceded by a minor peak between October and November. Seasonal fluctuations of diarrhoeal diseases were more pronounced in the north and inland provinces and less evident in the coastal areas (Figure S7 in the **Online Supplementary Document**).

The proportion of children under five with diarrhoea diseases among total cases was relatively similar in the different provinces, ranging from 40% (Maputo Province) to 60% (Nampula), with an overall ratio of 48% (Figure S8, Panel A in the **Online Supplementary Document**). The proportion of children under five with malaria had a broader range: from 9% (Maputo City) to 44% (Manica), with an overall ratio of 35% (Figure S8, Panel B in the **Online Supplementary Document**).

### Comparison of model forecasts

**Figure 1:**
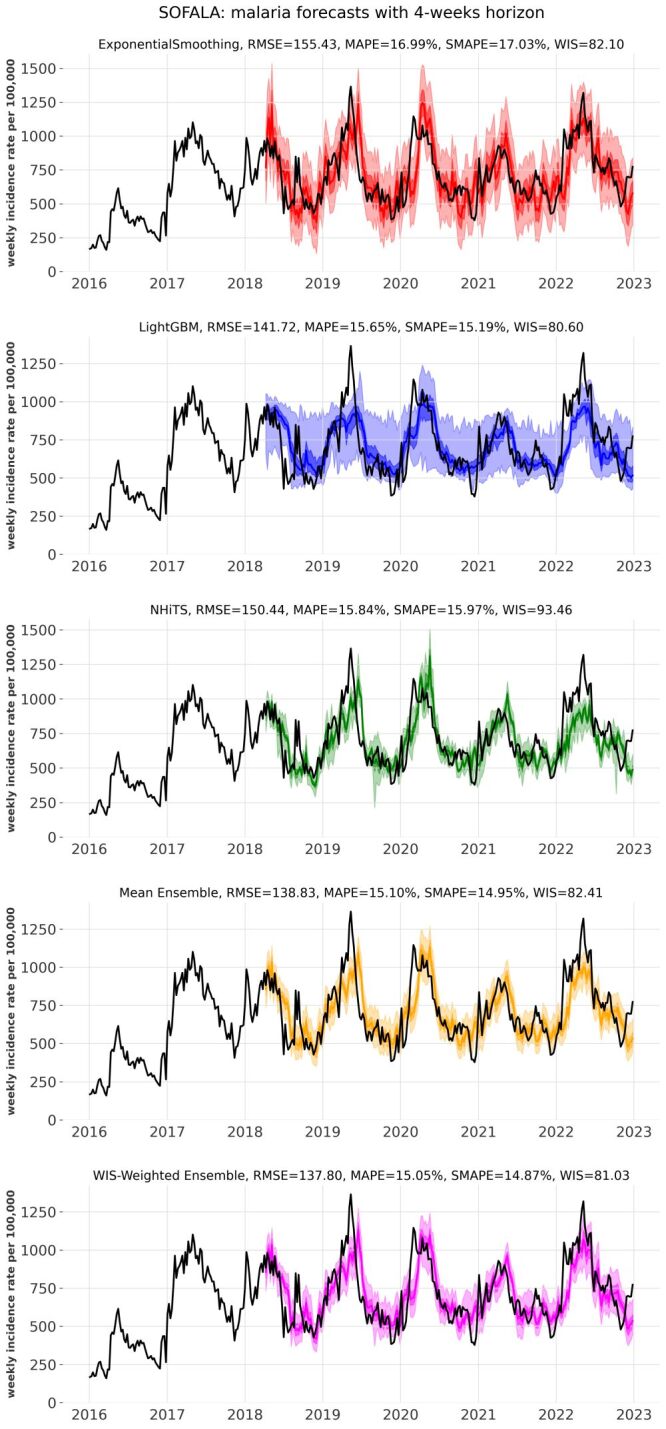
Forecast trajectories obtained through the rolling-origin evaluation procedure for the case of malaria incidence at Sofala province with a four-week forecasting horizon. Each subplot presents the forecasts of one of the tested models (Exponential Smoothing, LightGBM and N-HiTS) or model ensembles (mean ensemble and WIS-weighted ensemble). In each subplot the black line represents the data, coloured line shows the mean predictions, and the darker and lighter coloured regions show the 50% and 95% prediction intervals respectively. Predictions start from July 2018. The legend of each subplot contains prediction scores using the different metrics (RMSE, MAPE, SMAPE, WIS). Similar figures for all tested cases (malaria and diarrhoeal diseases incidence forecasts for all provinces and all forecast horizons) can be found online at: https://github.com/ramiyaari/Forecasting_Malaria_Diarrheal_Diseases_In_Mozambique/tree/main/figures/230624. LightGBM – Light Gradient Boosting Machine, MAPE – mean absolute percentage error, N-HiTS – Neural Hierarchical Interpolation for Time Series, RMSE – root mean squared error, SMAPE – symmetric mean absolute percentage error, WIS – weighted interval score.

**Figure 2:**
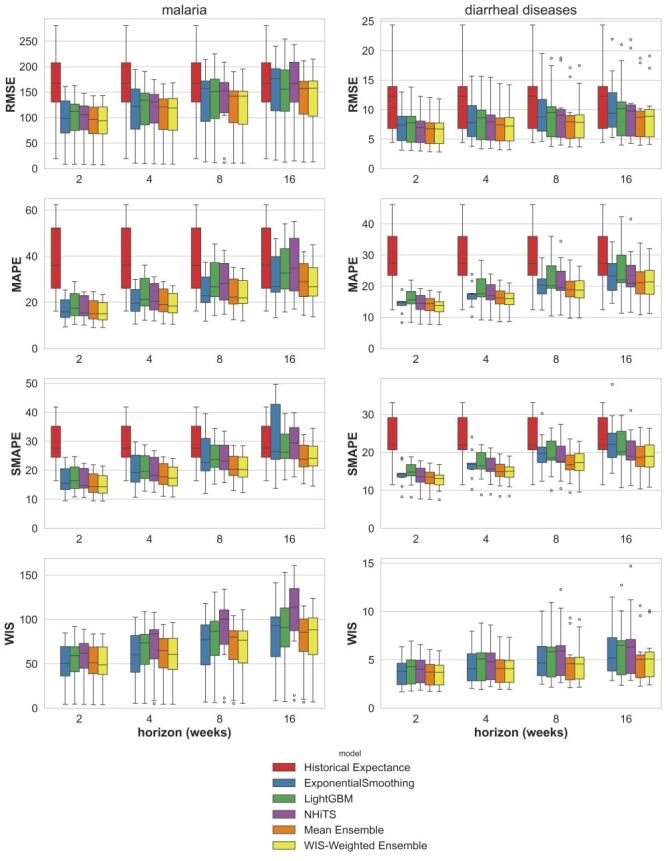
Summary of the forecasting models accuracy over all the tested cases using the different evaluation metrics. The variance depicted by the boxplots corresponds to the different provinces for which forecasts were made. Note that the WIS metric is not shown for the historical expectance forecasts as probabilistic forecasts were not derived for this model. WIS – weighted interval score.

**Figure 1** provides an example of the forecast trajectories obtained through the rolling-origin evaluation procedure using the tested models and ensembles. **Figure 2** presents a summary of all the results using the various evaluation metrics (Table S1–2 in the **Online Supplementary Document**), together with an evaluation of the historical expectance forecasts for comparison. The variance depicted by the boxplots derives from the forecasts obtained for the different provinces. The accuracy of the forecasts decreases as the forecast horizon increases. The different metrics all point to similar results. The forecasts generated with all tested models consistently outperformed the historical expectance for both disease systems and across all metrics for forecasting horizons of two, four, and eight weeks, and were either comparable to the historical expectance models or exhibited superior performance for the 16-week horizon. The three tested models (Exponential Smoothing, LightGBM, N-HiTS) exhibited comparable performance on average. When forecasting malaria incidence, the deep learning N-HiTS probabilistic forecasts gave narrow prediction intervals which lead to higher (less accurate) WIS scores. For both malaria and diarrhoeal diseases, the ensemble forecasts outperform the individual models’ forecasts, with the WIS-weighted ensemble slightly outperforming the mean ensemble model and providing the overall best forecasts (Figure S9 in the **Online Supplementary Document**).

Overall, forecasts for diarrhoeal diseases incidence prove more accurate than those for malaria incidence, as evident by the MAPE metrics. For malaria, the WIS-weighted ensemble yielded forecasts with a x̄ MAPE of 16% (range = 9–23% for the different provinces) for a two-week forecast horizon, 19% (range = 11–27%) for four weeks, 23% (range = 12–35%) for eight weeks and 28% (range = 14–45%) for 16 weeks. In comparison, historical expectance forecasts for malaria exhibit a x̄ MAPE of 38% (range = 16–62%). For diarrhoeal diseases, the WIS-weighted ensemble generated forecasts with a x̄ MAPE of 13% (range = 8–18%) for a two-week forecast horizon, 15% (range = 9–21%) for four weeks, 18% (range = 10–26%) for eight weeks and 21% (range = 11–32%) for 16 weeks. In comparison, historical expectance forecasts for diarrhoeal diseases have a x̄ MAPE of 29% (range = 12–46%). We note that similar results are obtained using the SMAPE metric, as the MAPE and SMAPE values for the WIS-weighted ensemble forecasts were highly correlated (Figure S10 in the **Online Supplementary Document**).

### WIS-weighted ensemble model forecasts at provincial level

**Figure 3:**
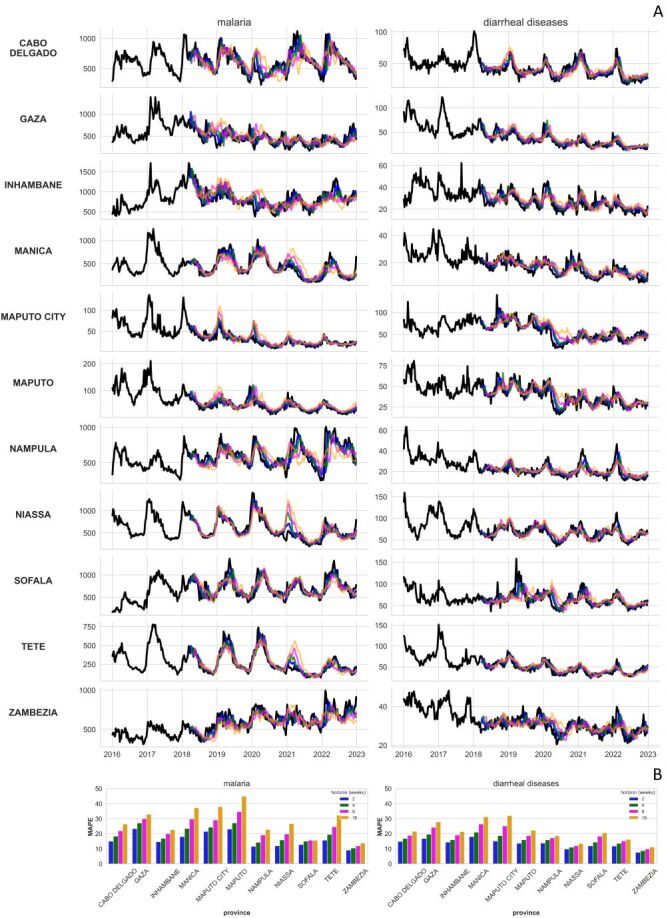
Forecast trajectories and evaluation of the WIS-weighted ensemble.** Panel A**. Incidence rates per province for malaria and diarrhoeal diseases (black) together with forecast trajectories of the WIS-weighted ensemble for the various forecast horizons (blue: two weeks, green: four weeks, magenta: eight weeks, orange: 16 weeks). **Panel B**. The calculated MAPE value for the forecast trajectories in Panel A. MAPE – mean absolute percentage error, WIS – weighted interval score.

**Figure 3**, Panel A presents the forecasts provided by the WIS-weighted ensemble model through the rolling-origin evaluation procedure for both malaria and diarrhoeal diseases, across each of Mozambique’s provinces and for each of the forecast horizons. **Figure 3**, Panel B provides the corresponding MAPE metrics for these forecasts. For both malaria and diarrhoeal diseases, the most accurate metrics were provided for the province of Zambezia with errors of 9, 10, 12 and 14% for malaria, and errors of 8, 9, 10 and 11% for diarrhoeal diseases, across forecast horizons of two, four, eight and 16 weeks respectively. The province of Maputo obtained the least accurate metrics for malaria with errors of 23, 27, 35 and 45% for malaria, while the province of Manica obtained the least accurate metrics for diarrhoea with errors of 18, 21, 26 and 31%, across forecast horizons of two, four, eight and 16 weeks respectively.

Examining the performance of the WIS-weighted ensemble model long-term forecasts in each year (Figure S11 in the **Online Supplementary Document**), reveals some years where forecast accuracy was very poor compared to other years in the same province. Specifically, poor malaria forecasts were obtained for the provinces of Cabo Delgado, Maputo and Maputo City during 2020, as well as for Manica, Niassa and Tete during 2021. Poor forecasts for diarrhoeal diseases were obtained for Maputo and Maputo City in 2020. In these provinces and years, the observed incidence deviated the most from the historical expectance projections (Figure S3 in the **Online Supplementary Document**).

### Visualisation dashboard

Figure S14 in the **Online Supplementary Document** is a screenshot from the visualisation dashboard. The dashboard features predictions for case numbers and rates of malaria and diarrhoeal disease presented on a province-level map of Mozambique, as well as a summary table and time series plots that include trends at the province levels. A demonstration version of the dashboard with forecasts made at epidemiological week 52 of 2022 can be accessed at: https://rodrigozepeda.github.io/MAQUINA/.

## DISCUSSION

A consistent and reliable surveillance system is the first step in building capacity for disease modelling and forecasting. Recently, infectious diseases surveillance and response has advanced considerably in Mozambique, with national agencies (Ministry of Health, INS) and international partners (Centres for Disease Control and Prevention, President’s Malaria Initiative, United States Agency for International Development) focusing on strengthening the country’s health system. Since 2016, SIS-MA aggregates incidence data for several disease systems (malaria, measles, diarrhoeal diseases, meningitis, and ‘febrile illness’ – a high-fever illness with unspecified cause) from over 150 districts across the country [27]. Here, in collaboration with researchers at INS, we have leveraged the SIS-MA surveillance data system to build a large-scale forecasting model for malaria and diarrhoeal diseases, two major contributors of morbidity and mortality in Mozambique, particularly among children under five. This is the first work that integrates forecasting models with routine disease surveillance in Mozambique. We developed an automatised framework that is used in conjunction with the SIS-MA data collection, is integrated into the INS infrastructure, and is complemented with a visualisation dashboard to streamline the sharing of information across different entities of the Mozambique Public Health system.

Our framework leverages an existing python library called Darts. We have selected three fast running models that are representative of three main statistical forecasting model types: a traditional statistical model, a machine-learning model and a neural-network model. The accuracy of the forecasts produced by the three models plus two multi-model ensembles, was compared to historical expectance from the previous years. The models outperformed historical expectance forecasts for both disease systems across forecast horizons of up to eight weeks and demonstrated comparable or superior performance for the 16-week horizon. Moreover, the multi-model ensembles consistently outperformed the individual models, confirming a trend shown in several previous forecasting efforts [12].

Using a WIS-weighted ensemble for diarrhoeal diseases we obtained forecasts within 13–21% of the observed incidence on average, for forecast horizons of 2–16 weeks, well below the 29% error on average of historical expectance forecasts. For malaria, the ensemble produced forecasts within 16–28% of the observed incidence on average for the same forecast horizons, which are well below the 38% error on average of historical expectance forecasts. The differences in forecast accuracy between provinces and between malaria and diarrhoeal diseases are related to the amount of variation in the forecasted time series, as measured by the coefficient of variation (COV) (Section G in the **Online Supplementary Document**). Time series with higher COV values tend to have lower forecast accuracy, explaining why malaria, which generally exhibits higher variation, has higher MAPE values compared to diarrhoeal diseases. This relationship is further demonstrated by the COV explaining 60–65% of the variance in MAPE values across provinces (Figure S12 in the **Online Supplementary Document**). For example, Zambezia province has the lowest COV for both malaria and diarrhoeal diseases, which corresponds to its relatively low MAPE values, whereas Maputo and Manica provinces exhibit high COV values for malaria and diarrhoeal diseases, respectively, corresponding to their higher MAPE values. The higher variation observed in malaria time series incidence may be related to the greater sensitivity of malaria to climatic conditions compared to diarrhoeal diseases. These differences between provinces may also derive from varying magnitudes of climatic fluctuation across regions.

By comparing predictions’ accuracy across years, we noted that the years displaying the lowest performance were 2020 and 2021, especially for malaria. Our time series overlaps with the Covid-19 pandemic beginning in 2020. The effect of quarantines and other social distancing measures as well as possible changes to the reporting efforts could have possibly led to an a-typical drop in the incidence of malaria and diarrhoeal diseases during this period, which may have corrupted forecast accuracy, particularly for longer horizons. In addition to the pandemic, armed insurgencies in Cabo Delgado disrupted the continuity of surveillance in that region with 20% of districts ceasing reporting incidence data between 2020–2021 (Figure S1 in the **Online Supplementary Document**).

A limitation of our data set is its relatively short length, as data prior to 2016 were not available. Deep learning models for time series forecasting typically perform better with longer data sets. However, even with this limited data set, the deep learning model N-HiTS performed just as well as the traditional statistical Exponential Smoothing model. As more data becomes available, deep learning approaches may become increasingly advantageous compared to traditional statistical models.

Both malaria and diarrhoeal diseases are modulated by climate patterns (temperature, precipitation and humidity) [28–31]. In this analysis we did not explicitly incorporate climatic data, for the sake of simplicity and operational ease. In addition, we did not consider spatial correlations between adjacent provinces, though by fitting global models to the provinces data (as we did with the LightGBM and N-HiTS models that support this option) (Section B in the **Online Supplementary Document**) we did enable the models to learn complex dependencies and correlations between provinces (but not in a spatially informed manner). As this study exclusively employs statistical models, the framework presented here can be readily adapted with minimal modifications to other disease systems exhibiting seasonal variations. Furthermore, this adaptable approach offers a scalable solution that can be applied in other low- and middle-income countries, enabling more effective public health responses to a wide range of infectious diseases.

## CONCLUSIONS

This study demonstrates the potential of disease incidence forecasting to support public health decision-making in Mozambique. Short-term forecasts (*e.g*. two and four weeks) can provide actionable insights for immediate interventions, such as allocating resources, managing outbreaks, and planning health care capacity, while mid- to long-term forecasts (*e.g*. eight and 16 weeks), though less reliable, can inform strategic planning for seasonal peaks or intervention scenarios. To enhance the practical application of these forecasts, we have developed a visualisation tool designed to present results in an accessible and actionable format. Future work will focus on investigating how this tool is adopted and used within Mozambique's public health system and identifying ways to improve its adaptability to local needs. Additionally, ensuring the consistent accuracy of the forecasting models in future applications will be crucial for building trust and integrating forecasts into public health decision-making. Addressing these priorities will help maximise the impact of forecasting in improving public health outcomes in resource-limited settings.

## Additional material


Online Supplementary Document
