# Supplementary material for: Infectious disease forecasting to support public health: use of readily available methods to predict malaria and diarrhoeal diseases in Mozambique
Source: J Glob Health. 2025 Apr 11;15:04114. doi: 10.7189/jogh.15.04114 (PMC11997905; doi:10.7189/jogh.15.04114)
Supplement: Online Supplementary Document [file jogh-15-04114-s001.zip › jogh-15-04114-s001.pdf]

Supplement to: Yaari A, Galanti M, Zepeda-Tello R, Chicumbe S, Jani I, Cassy A, et al. Infectious disease forecasting to support public health: use of readily available methods to predict malaria and diarrheal diseases in Mozambique. J Glob Health. 2025;15:04114.

Rami Yaari[1], Marta Galanti[1], Rodrigo Zepeda Tello[1],

Sergio Chicumbe[2], Ilesh Jani[3], Annette Cassy[3], Ivalda Macicame[2], Naisa Manafe[2],

Shannon M Farley[1,4], Wafaa M. El-Sadr[1,4], Jeffrey Shaman[1,5]

[1] Mailman School of Public Health, Columbia University, New York, NY

[2] *Instituto Nacional de Saúde* (INS), Maputo, Mozambique

[3]Ministry of Health, Maputo, Mozambique

[4] ICAP, Columbia University, New York, NY

[5] Climate School, Columbia University, New York, NY

Corresponding author: Rami Yaari (ry2460@cumc.columbia.edu)

A. <u>The forecasting models:</u>

a. **Exponential Smoothing**: An implementation of the Holt Winter's Exponential Smoothing method [1] utilizing exponentially weighted moving-average smoothing with both linear and seasonal trends. The seasonal periods were configured to span 52 weeks, representing an annual cycle. This model can only be run as a local model – training and forecasting each province time series separately.

b. **Light Gradient Boosting Machine (LightGBM)**: An implementation of the Gradient Boosting Decision Tree algorithm [2]. Gradient Boosting Decision Tree is an ensemble learning technique where multiple decision trees are sequentially trained to rectify the errors of their predecessors. It involves combining the predictions of individual trees, with each tree trained to minimize the residual errors of the preceding ones. LightGBM introduces various optimizations in data sampling and feature reduction, resulting in faster convergence and reduced memory usage compared to earlier implementations. The number of lag features to use, set in the configuration of the model, was determined by the number of significant lags in the partial auto-correlation plots of the time series. This model can be run both as a local model and a global model – training and forecasting all the provinces time series simultaneously.

c. **Neural Hierarchical Interpolation for Time Series** (**N-HiTS):** The N-HiTS forecasting model is a new state-of-the-art neural-network architecture tailored for time series forecasting [3]. It is an extension of N-BEATS, acronym for Neural Basis Expansion Analysis and Synthesis [4]. N-BEATS employs basis expansion to augment its training data and model non-linear relationships, as it searches for the optimal data

augmentation mixture for providing accurate forecasts. Its architecture was designed to achieve flexibility and scalability for application to various domains without the need for modifications, as well as fast training. The N-HiTS model introduces some variations to the architecture of N-BEATS that aims to enhance prediction stability and manage the computational complexity of model training, particularly addressing challenges associated with long forecast horizons. Like LightGBM, this model can be run as both a local and a global model.

B. Parameter tuning:

To set the parameters for the tested models we ran the rolling-origin evaluation procedure for the period between July 2018 until July 2019 with different combinations of parameter settings for each model and evaluated their performance. We selected the combination of parameters that lead to the best overall fit for all tested forecast horizons and employed them in the rolling-origin evaluation procedure for the whole time series (up to the end of 2022). Table SB1 depicts the parameters evaluated for each model and the selected parameter values. Only in one case (input_chunk_length of N-HiTS model) did we obtain different values for diarrheal diseases and malaria. For more information on the parameterization of each model see the Darts API models page at https://unit8co.github.io/darts/generated_api/darts.models.forecasting.html.

Machine learning and the neural network forecasting models in Darts can be run either locally or globally. Running locally means the model is trained on and produces forecasts for only one (univariate) target time series at a time. Running globally means that the model is trained on and produces forecasts for multiple (multivariate) target time series simultaneously. In our case, running a model locally means forecasting each province time series separately, while running

globally means forecasting all provinces simultaneously. For models that can be employed both locally and globally (i.e. LightGBM and N-HiTS), we assessed which option produced more accurate forecasts. We found that both LightGBM and N-HiTS performed better when run as global models (in addition to running much faster) and therefore used both as global models in our analysis.

Model	Tested Parameters	Selected Parameters
Exponential Smoothing	1) trend: [Additive, Multiplicative]<br>2) damped: [True, False]<br>3) seasonal: [Additive, Multiplicative]<br>8 combinations tested overall	trend=Additive<br>damped=True<br>seasonal=Multiplicative
LightGBM	1) output_chunk_length: [1,4,8]<br>2) lags_past_covariates: [1,2]<br>3) lags_future_covariates: [1,2]<br>12 combinations tested overall	output_chunk_length=1<br>lags_past_covariates=2<br>lags_future_covariates=2
N-HiTS	1) input_chunk_length: [12,26,52]<br>2) output_chunk_length: [1,4,8]<br>3) num_stacks: [1,3,5]<br>4) num_blocks: [1,2]<br>5) dropout: [0.1,0.3,0.5]<br>162 combinations tested overall	input_chunk_length=<br>52(diarrhea), 26(malaria)<br>output_chunk_length=1<br>num_stacks=5<br>num_blocks=2<br>dropout=0.1

Table SB1: Tested and selected parameters for each of the employed forecasting models.

C. Probabilistic forecasts:

Probabilistic forecasts in Darts are represented as samples, describing the joint distribution over time and components (e.g. different provinces in a multivariate model). Different models can employ distinct mechanisms for obtaining these samples. In the case of Exponential Smoothing, samples are acquired by running simulations with randomly sampled error. For LightGBM and N-HiTS, the samples are derived through a likelihood function defined in the model configuration. In this case, the models predict the parameter of the designated likelihood function rather than forecasting the future values directly. Using the estimated likelihood parameters, the likelihood

function generates samples for future time series values. We configure the likelihood function of these models to quantile regression [5], meaning the models are trained using 'pinball loss' – a tilted absolute value loss function where for quantile τ, forecast $\hat{y}$ and observation y, the loss function is defined as:

$$L(y, \hat{y}) = \begin{cases} -\tau(y - \hat{y}) & y \geq \hat{y} \\ (1 - \tau)(\hat{y} - y) & y < \hat{y} \end{cases}.$$

We set the number of values to be sampled for each time point to 1,000. Using the set of samples given by a probabilistic forecast, the Darts library calculates the expected quantile statistic for any requested quantile. We evaluated the probabilistic forecasts using the following 23 quantiles: 0.001, 0.025, 0.05, 0.10, 0.15, 0.20, 0.25, 0.30, 0.35, 0.40, 0.45, 0.50, 0.55, 0.60, 0.65, 0.70, 0.75, 0.80, 0.85, 0.90, 0.95, 0.975, 0.999.

D. Covariates:

Most of the forecasting models in Darts have the capacity to incorporate covariates, which are additional external time series data aiding the forecasting effort. Some models exclusively support past covariates - observed data up to the forecast time (e.g., weekly measured temperatures and rainfall). Conversely, some models exclusively support future covariates – data known in advance and extendable through the forecast horizon (e.g., climatological information such as historically averaged weekly temperatures and rainfall). Certain models accommodate both past and future covariates. Here, we do not employ any external information beyond information related to the time axis. Specifically, we include as covariates the week and month of the year extracted from the time of the incidence data. The incorporation of these data in each model depends on the type of support it offers for covariates: Exponential smoothing lacks

support for covariates, LightGBM supports both past and future covariates and N-HiTS supports only past covariates.

E. Evaluation metrics:

The forecasts generated using the rolling-origin evaluation procedure were evaluated using several metrics. For point forecasts - given time series y_t of observed values and time series $\hat{y}_t$ of predicted values, both of length T, we computed the following metrics:

- **Root Mean Squared Error (RMSE)**: $\sqrt{\frac{1}{T}\sum_{t=1}^{T}(y_t - \hat{y}_t)^2}$

- **Mean Absolute Percentage Error (MAPE)**: $100\frac{1}{T}\sum_{t=1}^{T}\left|\frac{y_t-\hat{y}_t}{y_t}\right|$

- **Symmetric Mean Absolute Percentage Error (SMAPE):** $200\frac{1}{T}\sum_{t=1}^{T}\frac{|y_t-\hat{y}_t|}{|y_t|+|\hat{y}_t|}$

MAPE and SMAPE are useful as they quantify the error in terms of a percentage, which allows comparison of forecasts across different scales. As all the incidence time series in our data are consistently well above zero, there is no possible issue with division by zero.

To evaluate probabilistic forecasts we employed the **Weighted Intervals Score (WIS)** metric [6]. For time series y of observed values and probabilistic forecast F given in the form of quantiles, the interval score (IS) for prediction interval $(1-\alpha)\cdot 100\%$ is given by:

$$IS_\alpha = (u - l) + \frac{2}{\alpha}(l - y)\cdot 1(l > y) + \frac{2}{\alpha}(y - u)\cdot 1(y > u)$$

where u and l denotes the $\alpha/2$ and $1-\alpha/2$ quantiles of F. The WIS for K prediction intervals given by $\alpha_1, \ldots, \alpha_k$ and predictive median m, is computed as:

$$WIS = \frac{1}{K+1/2}\left(\frac{|y-m|}{2} + \sum_{k=1}^{K} \frac{\alpha_k}{2} IS_{\alpha_k}\right)$$

F. <u>Model ensembles:</u>

In addition to the individual results of the forecasting models, we present outcomes for two model ensembles – a mean ensemble that simply averages the forecasts of the tested models, and a WIS-weighted ensemble that computes a weighted average using weights determined by the WIS score of the models in the preceding weeks as follows: Given forecast $f_m(t,h)$ for model $m = 1 \ldots 3$ (Exponential Smoothing, LightGBM, N-HiTS), performed at week t with forecast horizon h, and given the WIS scores for the forecasts (with forecast horizon h) of each model for the b weeks up to week t - $WIS_m(t-b-1,h) \ldots WIS_m(t,h)$ – the WIS-weighted ensemble forecast $f_{we}(t,h)$ was calculated as follows:

$$w_m = \frac{1}{\sum_{t'=t-b-1}^{t} WIS_m\,(t',h)}$$

$$f_{we}(t,h) = \frac{\sum_{m=1}^{3} w_m f_m(t,h)}{\sum_{m=1}^{3} w_m}$$

We used $b = 4$ weeks but found the results to have little sensitivity to a reasonable range around this value. We also tested other functions for generating the weights out of the WIS scores and found little sensitivity to this.

G. Relation between variation in a time series and its forecast accuracy:

We have found that the differences in forecast accuracy between provinces and between malaria and diarrheal diseases are related to the amount of variation in the forecasted time series: a time series with more variation will be harder to forecast than a stable time series. Figure S12 demonstrates this by plotting the coefficient of variation (COV) calculated for each time series (both malaria and diarrheal diseases incidence per 100,000 for every province) against the MAPE for forecasts generated using the WIS-weighted ensemble model. The time series' COV explains 60%-65% of the variance in the obtained MAPE values. Malaria time series tend to have more variation than diarrheal diseases time series, which explains the higher MAPE values obtained for malaria on average. In addition, Zambezia province has the lowest COV for both malaria and diarrheal diseases which explains its low MAPE values whereas Maputo the highest COV for malaria and Manica has the second highest COV for diarrhea which explains their high MAPE values for these conditions. The slope of the fitted regression lines (m) provide the expected link between timeseries COV and forecast accuracy given by MAPE. For the WIS-weighted ensemble model we obtain an increase of 0.26%, 0.31%, 0.40% and 0.57% in MAPE for each increase of 0.01 in the COV, for forecast horizons of 2, 4, 8 and 16 weeks respectively. Figure S13 shows results of similar regressions performed for all the tested models using the combined data of malaria and diarrheal diseases, displaying similar relation between a series' COV and its forecast accuracy. It provides another method for comparing the models. According to this analysis, the WIS-weighted ensemble provides the best results overall, followed by the mean ensemble, N-HiTS, Exponential Smoothing, LightGBM and Historical Expectance.

References:

1. Pan R. Holt–Winters Exponential Smoothing. Wiley Encycl Oper Res Manag Sci [Internet]. John Wiley & Sons, Ltd; 2010 [cited 2024 Mar 11]. Available from: https://onlinelibrary.wiley.com/doi/abs/10.1002/9780470400531.eorms0385

2. Ke G, Meng Q, Finley T, et al. LightGBM: A Highly Efficient Gradient Boosting Decision Tree. Advances in neural information processing systems. **2017**; 30.

3. Challu C, Olivares KG, Oreshkin BN, Ramirez FG, Canseco MM, Dubrawski A. NHITS: Neural Hierarchical Interpolation for Time Series Forecasting. Proc AAAI Conf Artif Intell. **2023**; 37(6):6989–6997.

4. Oreshkin BN, Carpov D, Chapados N, Bengio Y. N-BEATS: Neural basis expansion analysis for interpretable time series forecasting [Internet]. arXiv; 2020 [cited 2024 Jan 16]. Available from: http://arxiv.org/abs/1905.10437

5. Koenker R. Quantile Regression [Internet]. Cambridge: Cambridge University Press; 2005 [cited 2024 Jan 17]. Available from: https://www.cambridge.org/core/books/quantile-regression/C18AE7BCF3EC43C16937390D44A328B1

6. Bracher J, Ray EL, Gneiting T, Reich NG. Evaluating epidemic forecasts in an interval format. Pitzer VE, editor. PLOS Comput Biol. **2021**; 17(2):e1008618.

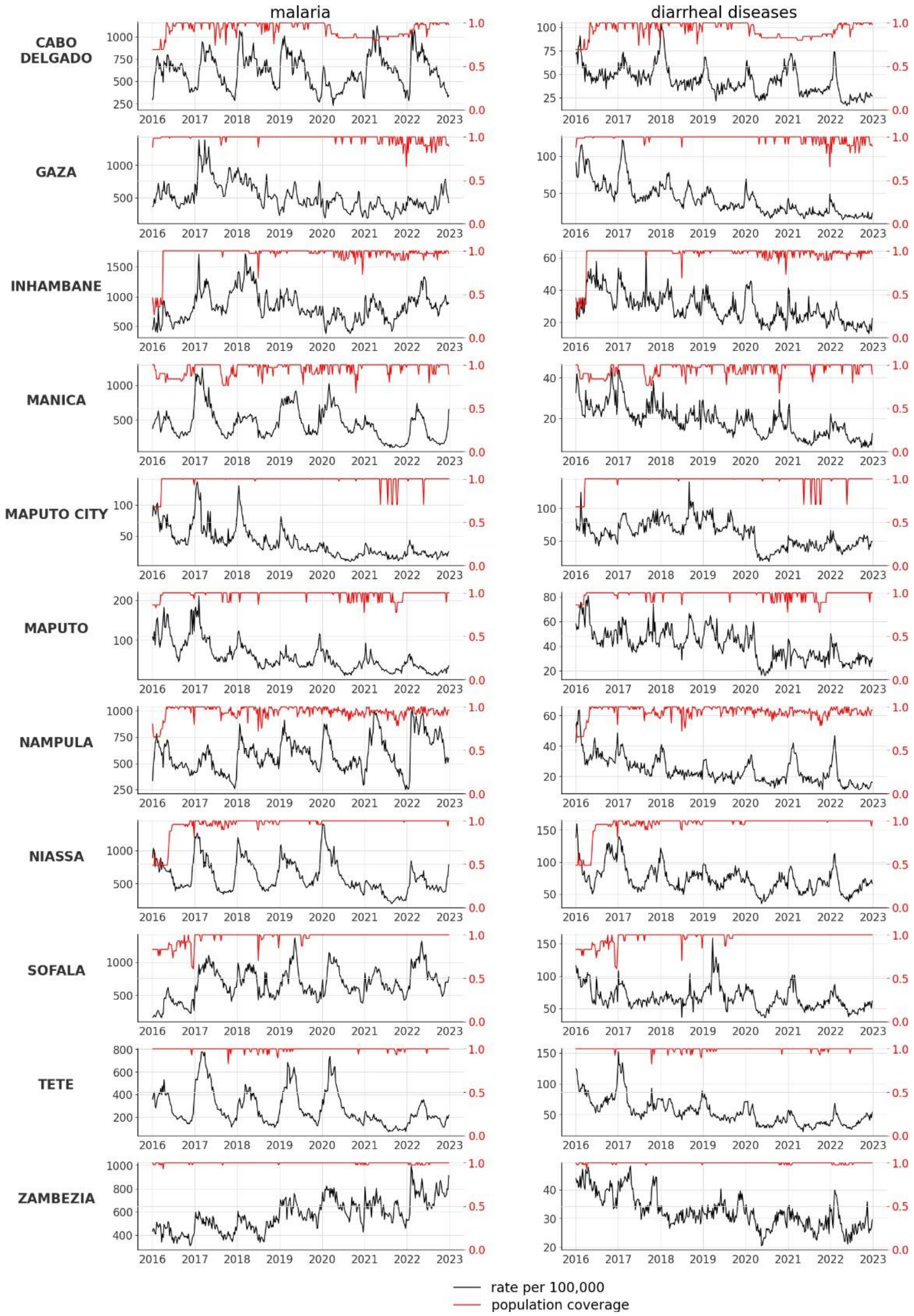

Figure S1: Weekly incidence rates of malaria and diarrheal diseases in each province (black - left y-axis) together with the weekly population coverage of the reports at the provincial level based on the districts reporting data (red – right y-axis).

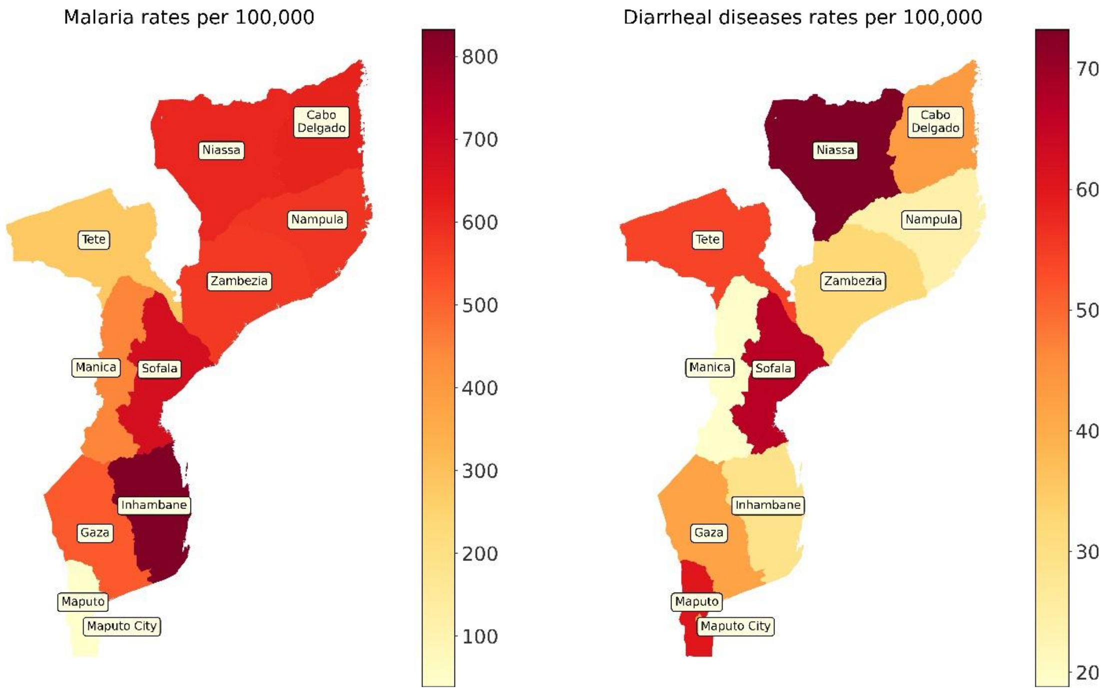

Figure S2. Maps depicting the provinces of Mozambique with the average weekly rates of malaria and diarrheal diseases per 100 000 population across the study period from 2016–2022.

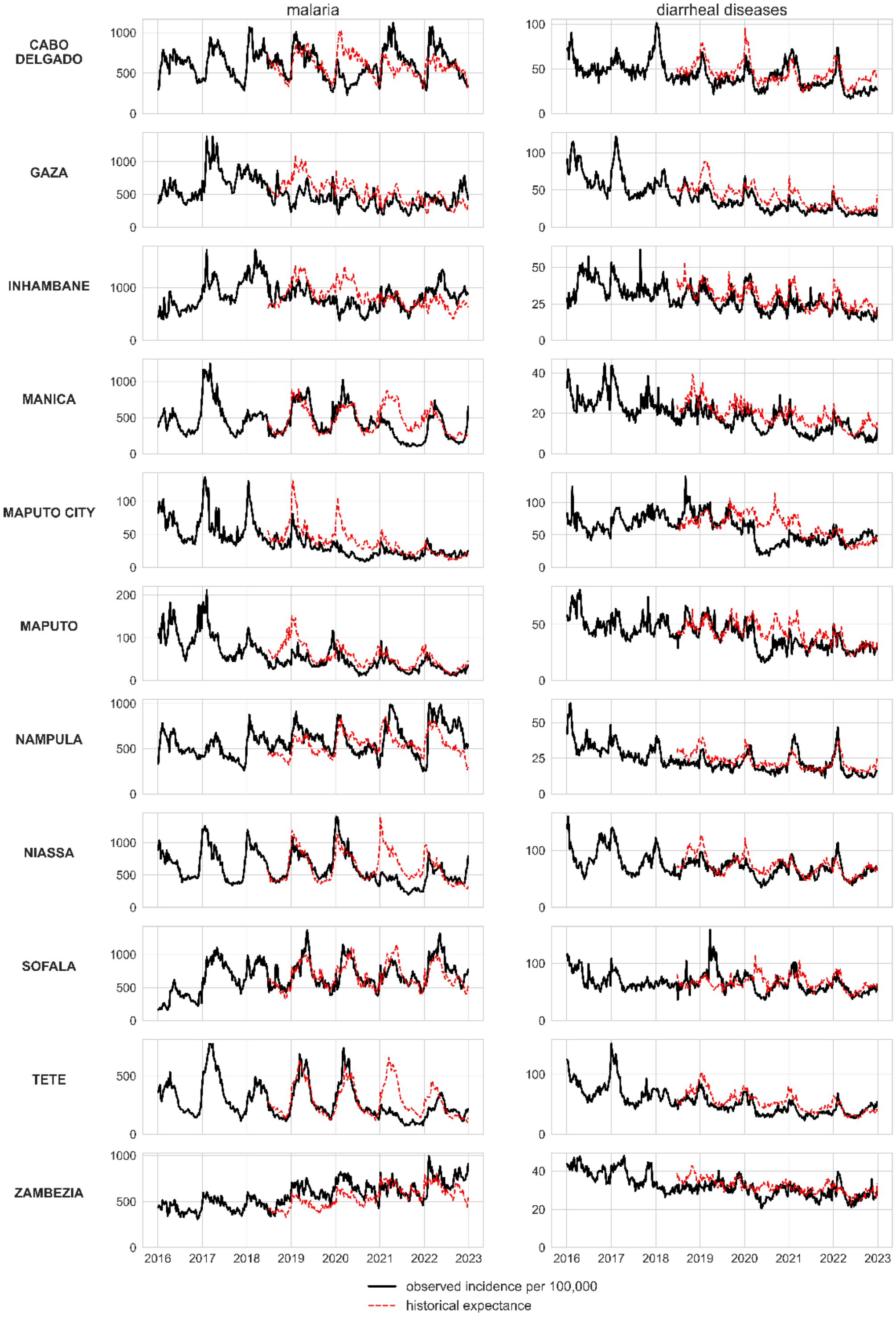

Figure S3. Time series of weekly incidence rates of malaria and diarrheal diseases per 100 000 population for each of Mozambique provinces, together with historical expectance projections based on a window of the past two years.

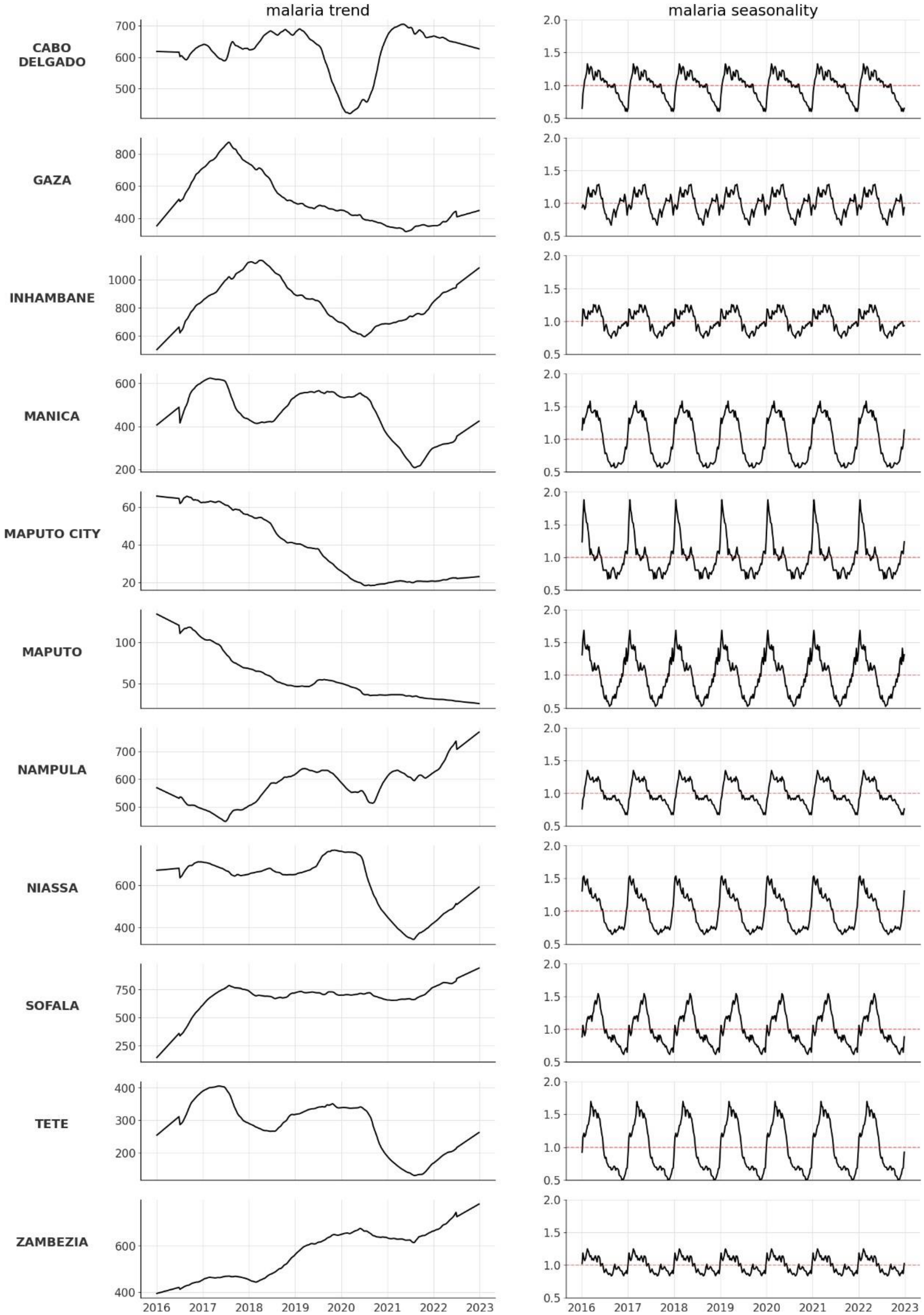

Figure S4: Decomposition of malaria weekly incidence per 100,000 population time series into a trend and a seasonal component for each province.

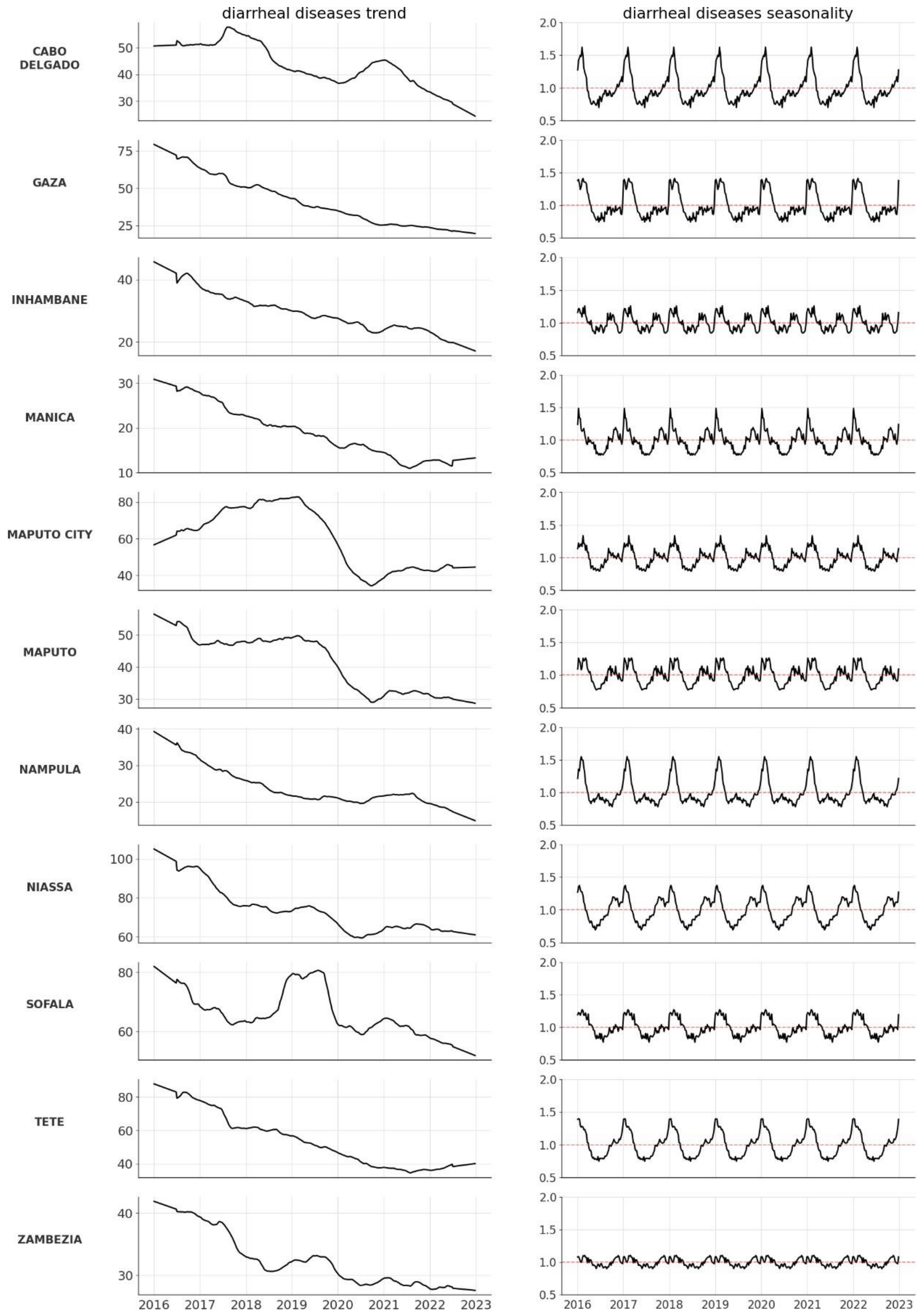

Figure S5: Decomposition of diarrheal diseases weekly incidence per 100,000 population time series into a trend and seasonal component.

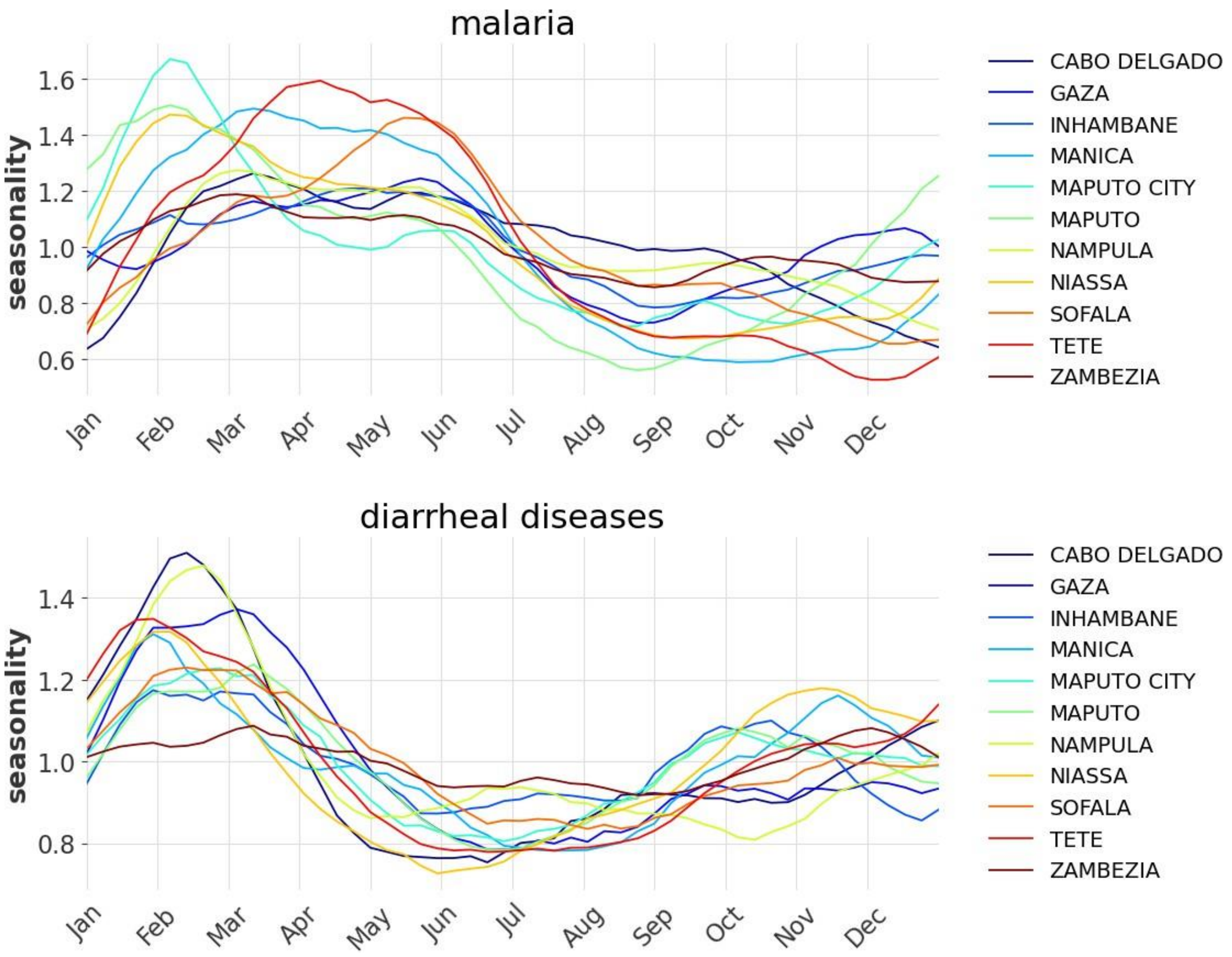

Figure S6: Comparison of malaria and diarrheal diseases seasonality at the province level. Y-axis are the seasonal component of each province (Figures S4-S5), smoothed using a moving-average with a 5-week window.

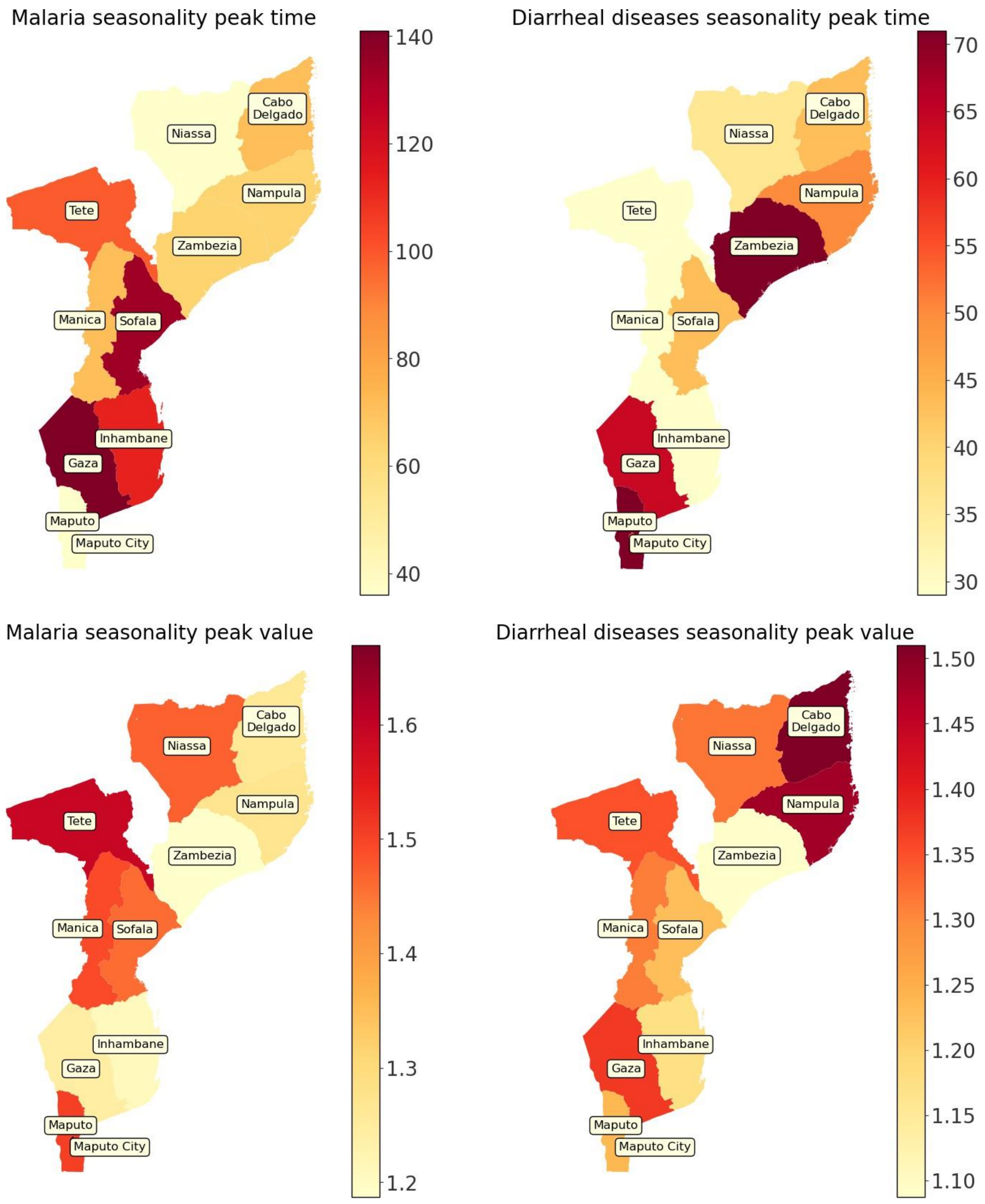

Figure S7: Maps showing a comparison of malaria and diarrheal diseases seasonality peak time and amplitude across the different provinces. Peak timing is measured in days from January 1st. The values for these maps were taken from Figure S6.

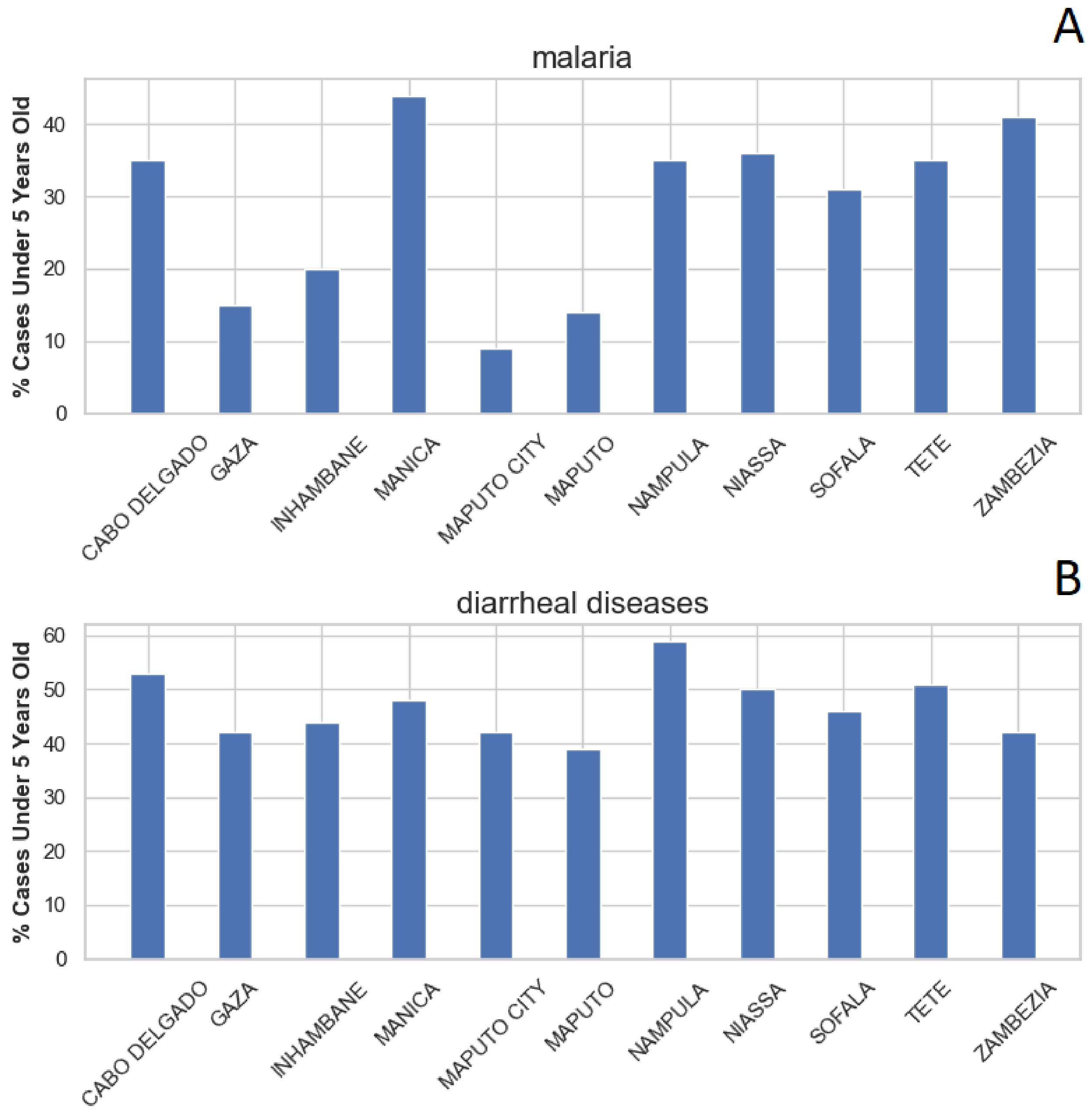

Figure S8: Percentage of malaria and diarrheal diseases cases among individuals under 5 years old in each province. Based on monthly data from the periods January 2019 - December 2022 (malaria) and January 2017 – June 2024 (diarrheal diseases).

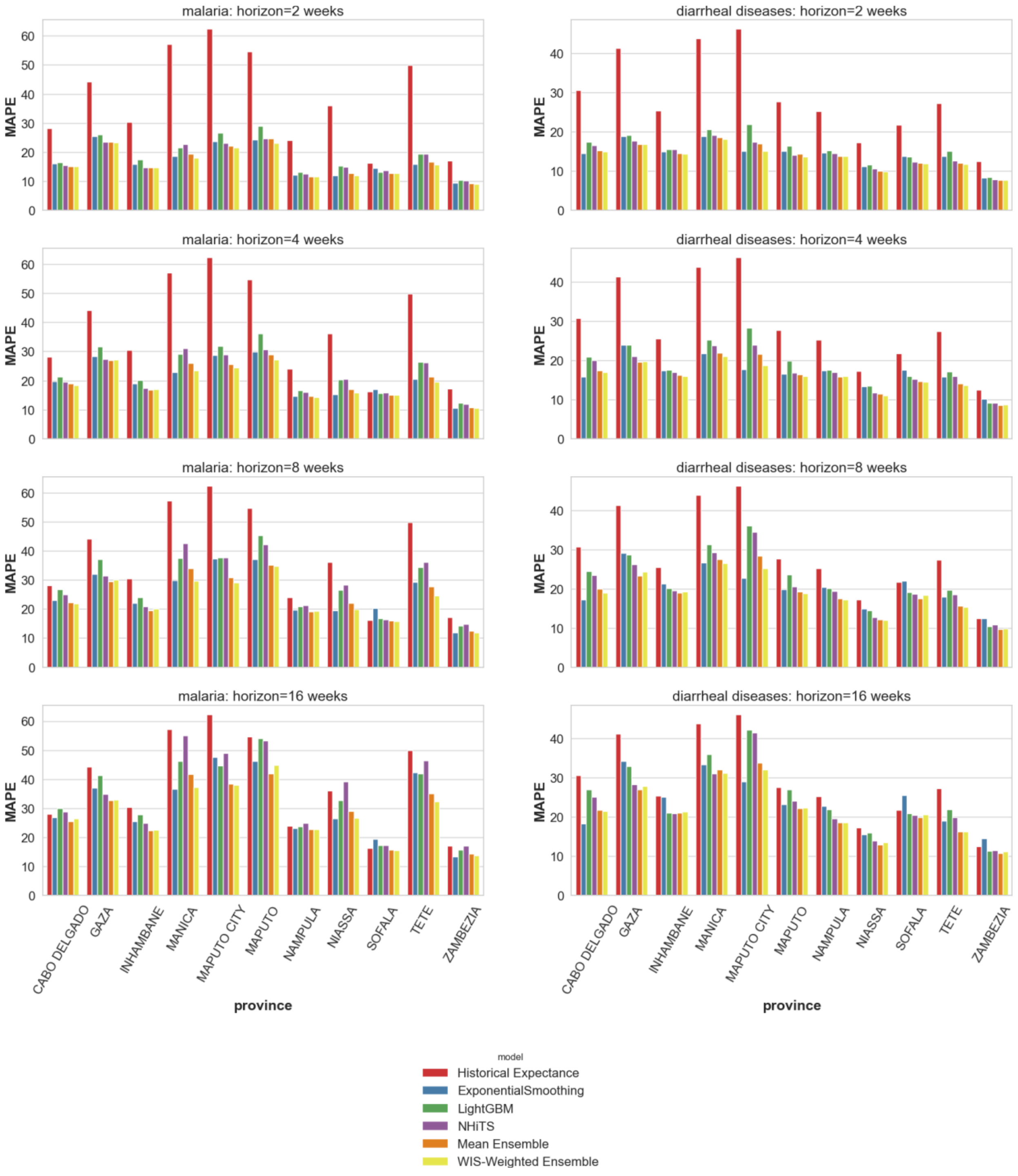

Figure S9: Accuracy of forecasts of the three tested models and the two model ensembles as given by the MAPE metric, for each forecast horizon and province.

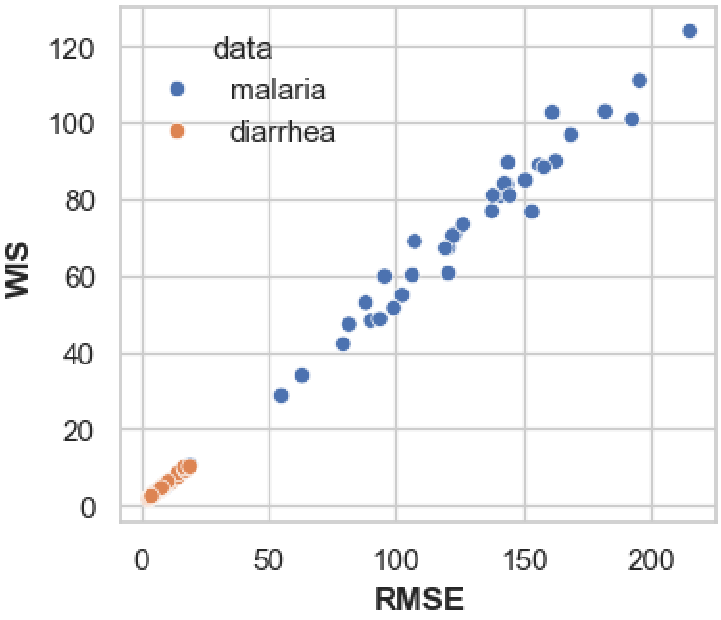

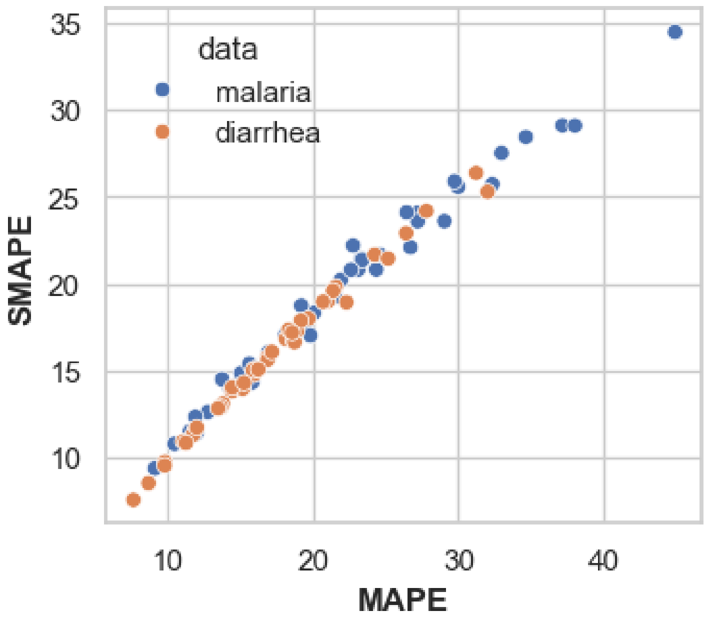

Figure S10: Scatter plots of RMSE vs WIS scores (top) and MAPE vs SMAPE scores (bottom) obtained for the WIS-weighted ensemble forecasts, broken down by type of data.

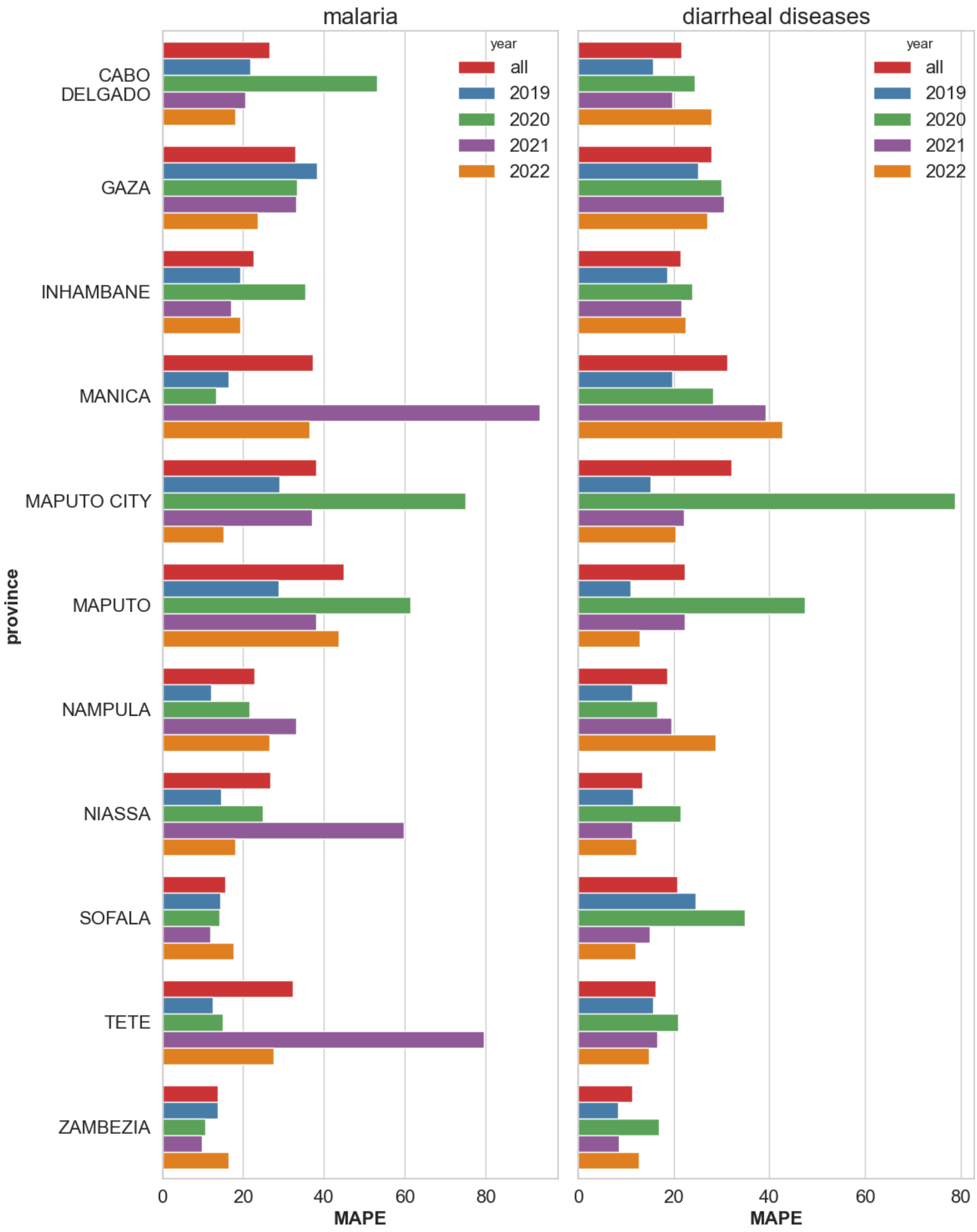

Figure S11: MAPE values of the WIS-weighted ensemble forecasts with forecast horizon of 16 weeks for each province in various years and overall.

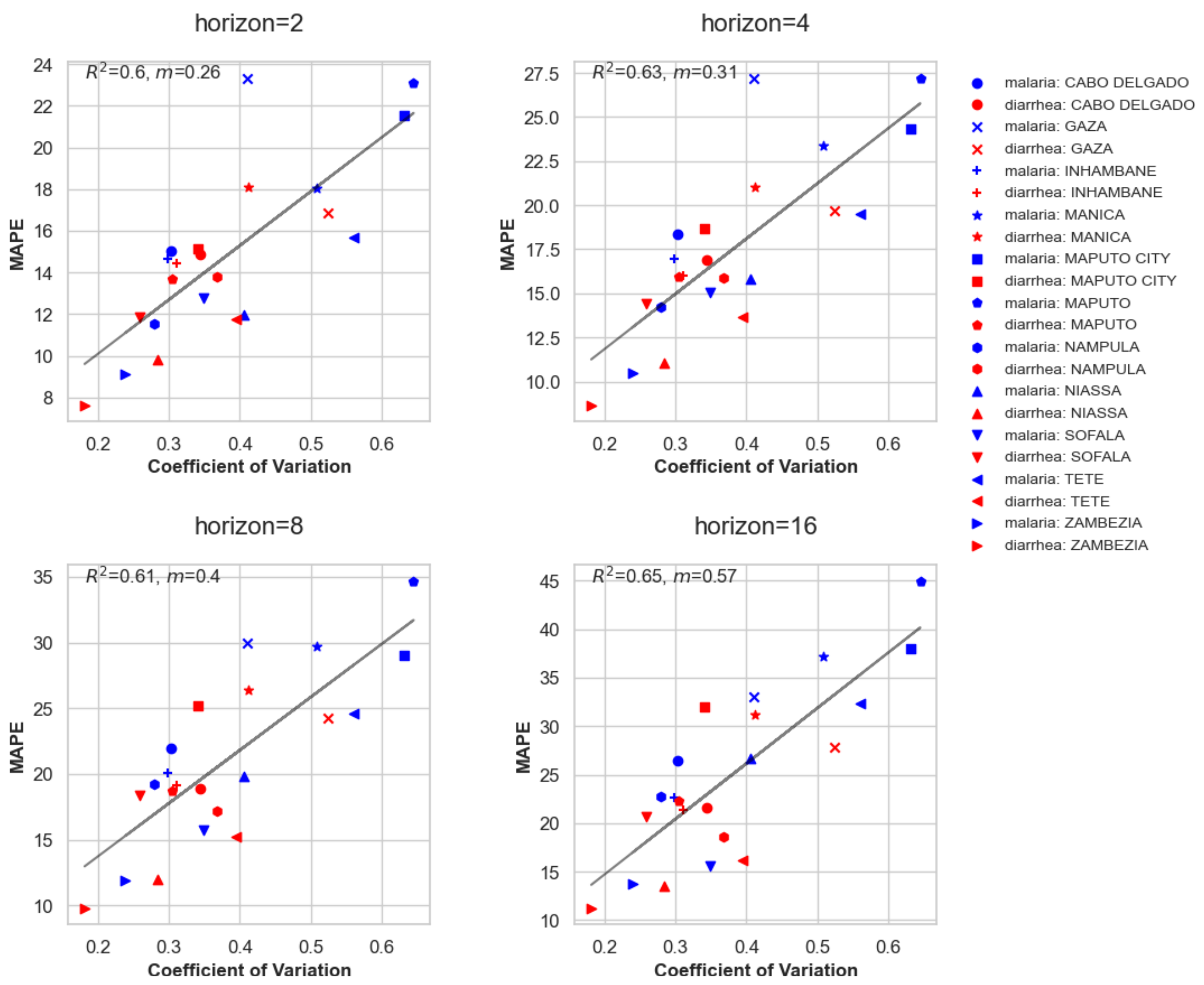

Figure S12: Scatter plots of the coefficient of variation of a time series against the obtained MAPE value of its forecasts using the WIS-weighted ensemble, for each of the forecast horizons. Also shown and given are the regression lines and their coefficient of determination (R^2) and slope (m), which provides the expected increase in MAPE for each 0.01 increase in the coefficient of variation.

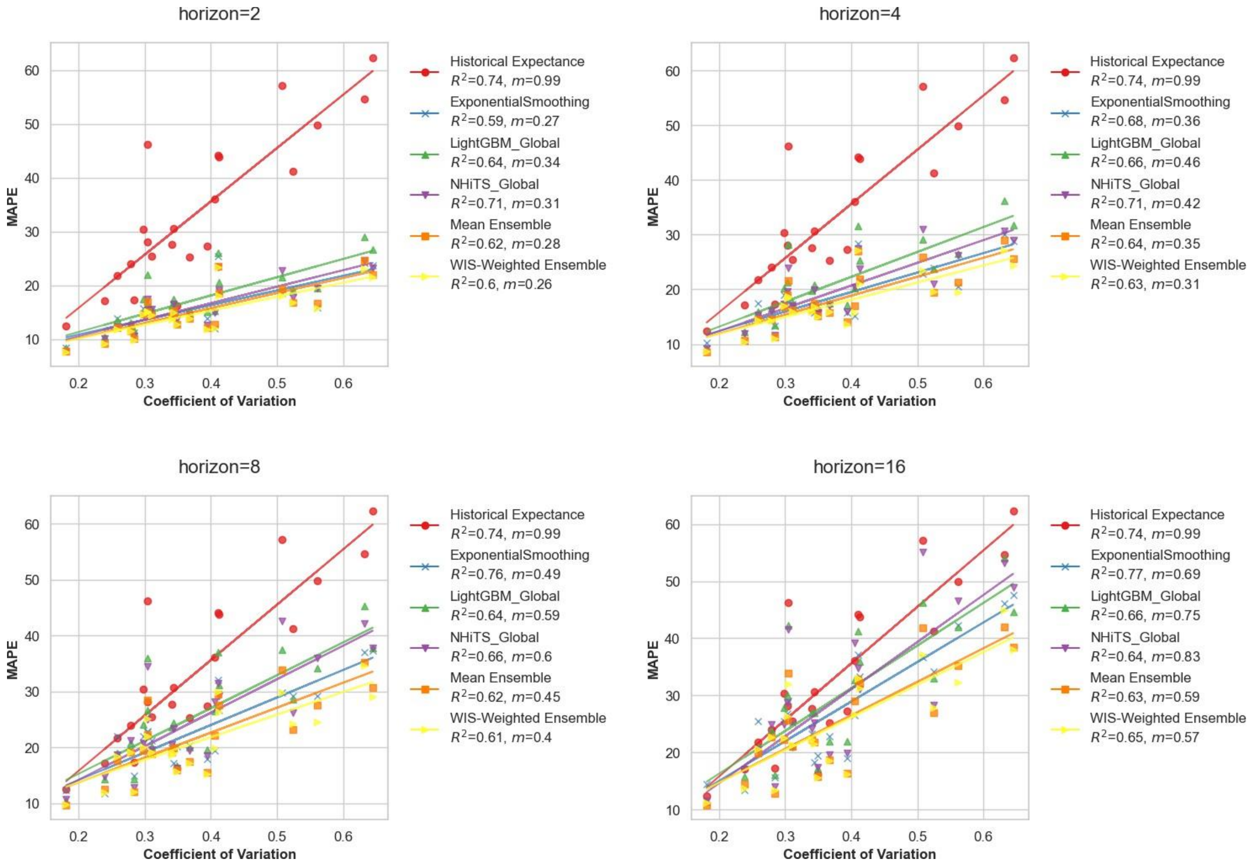

Figure S13: Scatter plots with regression lines of the coefficient of variation of a time series against the obtained MAPE value of its forecasts using each model, for each of the forecast horizons, using the combined data sets of malaria and diarrheal diseases.

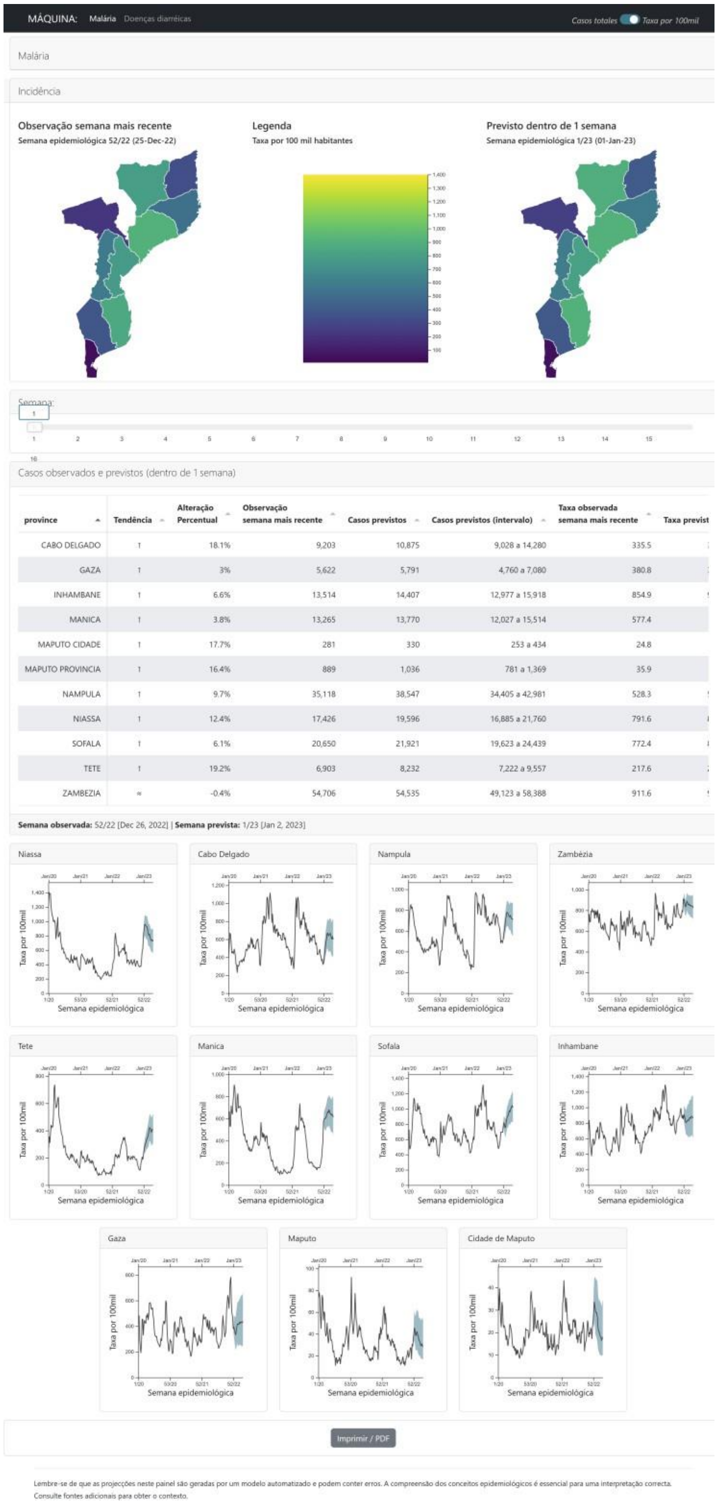

Figure S14. Screenshot of the model's dashboard showing the map and plots for the last epidemiological week of 2022.
